# The involvement of exosomes in the diagnosis and treatment of pancreatic cancer

**DOI:** 10.1186/s12943-020-01245-y

**Published:** 2020-08-27

**Authors:** Abakundana Nsenga Ariston Gabriel, Fang Wang, Qinlian Jiao, Umwali Yvette, Xuemei Yang, Samed Ahmed Al-Ameri, Lutao Du, Yun-shan Wang, Chuanxin Wang

**Affiliations:** 1 Department of Clinical Laboratory, The Second Hospital, Cheeloo College of Medicine, Shandong University, Jinan, Shandong 250033 People’s Republic of China; 2 Department of Clinical Laboratory Diagnostics, School of Medicine, Cheeloo College of Medicine, Shandong University, Jinan, Shandong 250012 People’s Republic of China; 3 Institute of Medical Sciences, The Second Hospital, Cheeloo College of Medicine, Shandong University, Jinan, Shandong 250033 People’s Republic of China; 4 Marine College, Shandong University, Weihai, 264209 People’s Republic of China; 5 Department of Clinical Laboratory, Qilu Hospital, Cheeloo College of Medicine, Shandong University, Jinan, Shandong 250012 People’s Republic of China

**Keywords:** Exosomes, Pancreatic cancer, Biomarker, Treatment

## Abstract

At the moment, pancreatic cancer is among the deadliest gastrointestinal diseases, and pancreatic cancer growth is a complex biological process that is based on different kinds of genes. Exosomes are extracellular vesicles containing microRNAs (miRNAs), messenger RNA (mRNA), and proteins, they act as the most prominent mediator of intercellular communication, and they regulate, instruct, and re-educate their surrounding microenvironment and target specific organs. Due to accumulative evidence proved that exosomes are involved in metastasis, cell proliferation, EMT, angiogenesis, and TME of pancreatic cancer, exosomes are crucial potential candidates to detect pancreatic cancer early. This review aims to convey the current understanding of the main functions employed by exosomes in early diagnosis and treatment of pancreatic cancer.

## Introduction

At the moment, pancreatic cancer is among the most deadly gastrointestinal cancerous diseases [1]. Pancreatic cancer symptoms are atypical, the development of the illness is too fast, and there are no sensitive early diagnostic biomarkers or proper clinical treatment [2]. Pancreatic cancer growth is a complex biological process that is based on different genes that are mutated [3]. PC is also a malignant disorder with poor prognosis, listed globally for the fourth fatal malignancy [4]. Statistically, in the United States, there are nearly 45,000 reported new cases recorded yearly, and 5-year survival rates over are less than 5% [5]. Even though researches have achieved considerable advances in several aspects, especially genes, proteins, and cells, most of the PC malignant biological processes need to be explained at a very advanced level [6]. Many pancreatic cancer patients fail to present influential signs until they enter the stage of disease progression. Various studies reported some significant risk factors that may cause the development of pancreatic cancer. Some of those risk factors include smoking [7], family history of chronic pancreatitis [8], advancing age [9], male sex [10], diabetes mellitus [11], obesity [12], occupational exposures, African-American ethnic origin [13], a high-fat diet, diets high in meat and low in vegetables and folate [14], and possibly Helicobacter pylori infection [15]. Compared to many other cancer types, pancreatic cancer is remarkably caused by 4 essential genes that are mutated at a high number of patients suffering from PC. The most altered gene within pancreatic ductal adenocarcinoma consists of K-ras the proto-oncogene, which is usually mutationally activated above 90% cases [16]. Besides, tumor suppressors such as CDKN2A [17], p53 [18], and DPC4/SMAD4 [19] are also altered in above 95%, between 50 and 75, and 55% of cases, respectively [20]. Exosomes are extracellular vesicles containing microRNAs (miRNAs), messenger RNA (mRNA), and proteins. Accumulative pieces of information proved that exosomes are potential candidates for early detection and diagnosis of pancreatic cancer [21]. Currently, various studies showed that increased or decreased expression of exosomes play an important role in different kinds of cancer, including pancreatic cancer [22]. For instance, exosomes are stimulating factors for the initiation and the development of pancreatic cancer, and this shows the ability of exosomes to be used as diagnostic biomarkers of pancreatic cancer [22].

Moreover, it has also been reported that there is a vast difference between the expression level of tumor-derived serum exosomes between pancreatic cancer patients and non-pancreatic cancer patients [23]. The conditions as mentioned above are due to the fact of that during carcinogenesis, pancreatic cancer patients secrete high amounts of circulating exosomes [24], and these secreted circulating exosomes are significantly detectable in body fluids, this also made researchers consider exosomes as potential diagnostic tools for early detection of pancreatic cancer [25]. Besides, recent research demonstrated that exosomes could directly and specifically target the oncogenic KRAS, which is also an essential gene in the development of pancreatic cancer, this made exosomes to be the best novel therapeutic candidates for pancreatic cancer [26]. In general, pancreatic cancer lacks different early diagnostic biomarkers and molecular target treatment that can significantly help in the battle of reducing pancreatic cancer fatalities around the world. In this review, we focus on the recent advances in our understanding of the main functions of exosomes in pancreatic cancer early diagnosis and treatment.

## Exosome main components

**Fig. 1:**
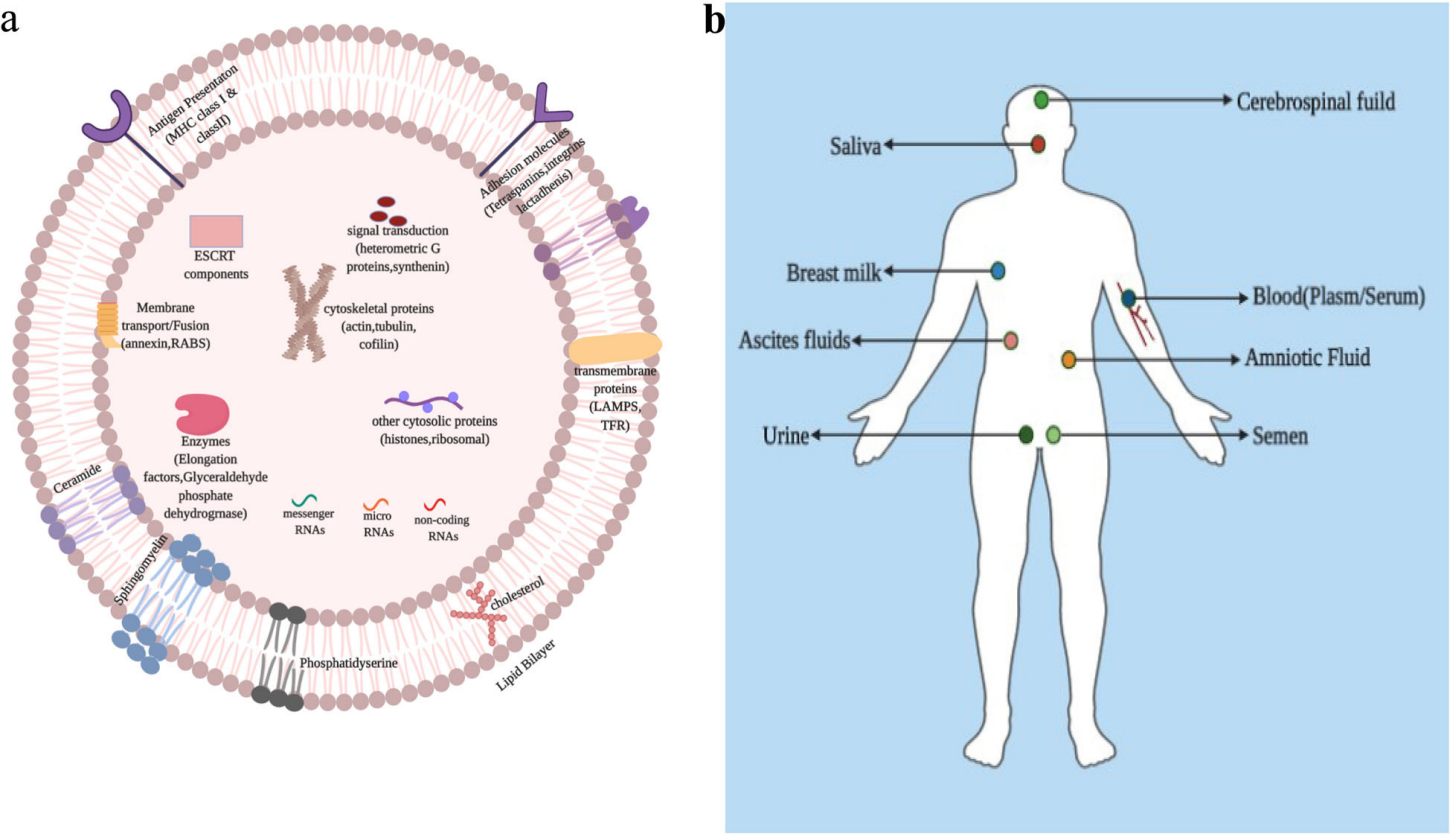
**a:** Shows the essential components of exosomes**. b:** Presents different boy fluids where exosomes are found

Exosomes are extracellular vesicles which are often formed and produced by several cells; their size varies around 30 to 100 nm in diameter, and they have bilayer lipid [27]. As components, exosomes are composed of proteins, DNA, mRNA, miRNA, lipids, and non-coding RNA [28], all of the elements mentioned above are portrayed in Fig. 1a. One of the main functions of exosomes is to transfer nucleic acids and proteins into various recipient cells, and this facilitates both the transport of essential substances and the communications between cells [29].

Exosomes are composed of a range of proteins, such as heat-shock proteins (Hsp70, Hsp90) [30], tetraspanins (CD9, CD81), proteins associated with ESCRT (Alix, Tsg101), cytoskeletal proteins (actin, tubulin), and GTPases [31]. These proteins participate in the production sorting and secretion of exosomes [32], and they are also involved in antigen presentation, membrane microdomain structure, cytoskeleton, and endosomal network [33].

Exosomes also comprise nucleic acids, such as DNA, mRNAs, miRNAs, and long non-RNAs (lncRNAs) [34]. Among these, miRNAs are indeed known molecules that are involved in gene regulation after genetic transcription events [35]. Growing evidence has shown that exosomal miRNAs are strongly linked to several cancers, including pancreatic cancer [36]. Different studies focused on exosomal microRNA because some exosomal contents are the same as that of the original tumor [37]. As it is presented on Fig. 1b, exosomes can be detected in different kinds of body fluids such as cerebrospinal fluid, saliva, plasma/serum, breast milk, ascites fluids, amniotic fluid urine and semen, which made exosomal microRNA to be considered as a candidate for diagnosing various cancers, including pancreatic cancer [38]. Additional exosomal components like cholesterols, ceramide, sphingomyelins are also crucially involved in the initial production of exosomes [39].

## Leading significant clinically relevant roles of exosomes in pancreatic cancer

### Exosomes play essential roles in pancreatic cancer cell proliferation, metastasis, EMT, and angiogenesis

**Fig. 2:**
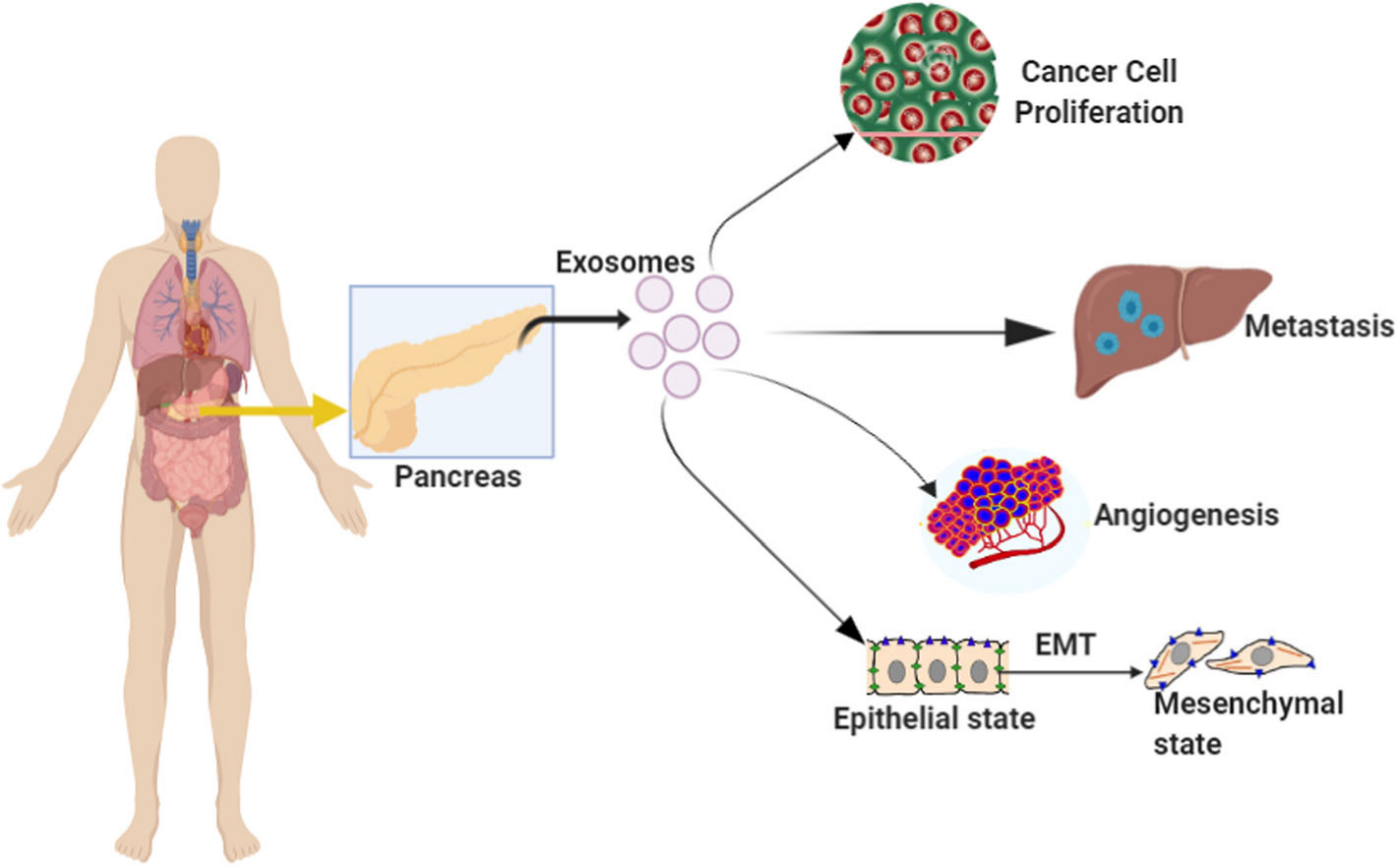
Highlights the roles of exosomes in pancreatic cancer progression that includes cell proliferation, metastasis, angiogenesis, and EMT

Exosomes have been shown to regulate both the development and progression of pancreatic cancer, which means that exosomes are involved in EMT, Metastasis, and cell proliferation Fig. 2 [40]. For example, Li Z et al. reported that tumor-generated exosomes miR-222 induced the invasion and proliferation of nearby tumor cells, and these outcomes are achieved by regulating and re-localizing P27 [41]. Botla et al. demonstrated that downregulating microRNA-192 expression increased in the progression of pancreatic cancer [42]. Besides another study conducted by Y. Fu and his colleagues indicated that the decreased expression of exosomal miR-98-5p promotes metastasis and cell proliferation of pancreatic cancer by downregulating MAP 4 K4. This exosomal microRNA also suppressed the MAPK/ERK pathway, which is essential in tumor formation [43]. Besides, Wang S et al. reported that exosomal miR-182 increased both cell proliferation and migration by targeting β-TrCP2, which plays a critical role in regulating cell cycle checkpoints. Also, they proved that exosomal miR-182 also plays a significant role in pancreatic cancer progression [44]. Metastasis is essential in the progress of different types of cancers, and various studies have reported that exosomes are currently involved in pancreatic cancer metastasis [45]. It has been reported that exosomal miR-301a-3p mediated M2 macrophage polarization via PTEN/PI3Kγ, and in return, this considerably promoted PC metastasis [46].

Interestingly, another study also reported that miR-21 expression in PDAC TAFs is very active in promoting both invasion and metastasis in pancreatic cancer [47]. Pancreatic cancer EMT is another critical, momentous event in pancreatic cancer development, and it has been found that it is possible to modulate EMT by using exosomes. For instance, miR-429, together with the low expression of miR-141, decreased pancreatic cancer development, metastatic behaviours, and it has also enhanced the expression rate of the primary regulators of EMT in pancreatic cancer [48]. A recent study by Hu J et al. reported that miR-361-3p regulated ERK1/2-induced EMT in pancreatic cancer by facilitating DUSP2 mRNA degradation [49]. Angiogenesis, which is the formation of the new blood vessels that are necessary to transport oxygen and other substances needed to grow or the spreading of the disease, angiogenesis is an essential event in different types of cancer, including pancreatic cancer [50]. Besides, accumulating shreds of evidence proved that exosomes play an essential role in pancreatic cancer angiogenesis [51]. Shang et al. reported that PC cell-derived exosomal microRNA-27a significantly stimulated the angiogenesis of HMVEC in pancreatic cancer through BTG2 [52]. In conclusion, exosomes affect pancreatic cancer progression by regulating essential features such as angiogenesis, EMT, cell proliferation, and metastasis.

### Exosomes are promising tools to be used as pancreatic cancer biomarkers

A cancer biomarker corresponds to a compound or process that shows the existence of cancer in the body [53]. A biomarker can be a substance naturally produced by a tumor or the body’s unique reaction to the presence of disease [54]. One of the studies carried out on cancer-related exosomes reported that cancer cells release lots of exosomes compared to non-cancerous cells. It has also been reported that cancer exosomes are crucially released into cancer microenvironment and circulatory system [55]. These results prove that exosome’s components, which include miRNAs, are considered as a promising screening tool, and they can be used to strengthen the responsiveness and reliability of early pancreatic cancer detection [56]. Xu Y-F et al. reported that miR-196a and miR-1246 were highly expressed in exosomes derived from pancreatic cancer. These two miRNAs were found to be slightly elevated in plasma exosome specimens from patients who had localized PC juxtaposed to patients who had not PC [57]. Besides, Goto et al. also reported that exosomal miR-191, exosomal miR-21, and exosomal miR-451a were substantially increased in patients with PC and IPMN which was not the case to the patients who did not have PC [58]. Abue et al. also noted that plasma exosomes miRNA-483-3p expression was higher in pancreatic cancer patients compared with IPMN patients [59]. These findings, as mentioned above, make exosomal miRNAs a sensitive tool for early diagnosis of pancreatic cancer and an essential biomarker for distinguishing pancreatic cancer from IPMN. Furthermore, it has also been reported that miRNA-16a and miRNA-196a, together with CA19–9 [60], provided a positive outcome towards the identification of stage I of PC [61], suggesting that these exosomal microRNAs can also be used as PC peripheral biomarkers [62]. Also, in Kawamura’s study, miR-4525, miR-451a, and miR-21 derived from portal vein have been proved possible biomarkers to determine the likelihood of recurrence and poor survival in patients with pancreatic cancer [63]. Furthermore, CKAP4, a DKK1 receptor, and secreted by exosomes, was also found to be a potential biomarker for pancreatic cancer [64]. Thus far, researchers did not stop exploring the role of exosomes as biomarkers for pancreatic cancer. By doing so, they found that exosomes extracted from pancreatic juice can catch a defined range of pathological biomarkers to help identify pancreatic cancer [65]. Machida et al. also reported that miR-1246 and miR-4644, which are freely available in saliva, could be considered as potential biomarkers for pancreaticobiliary tract cancer [66]. The studies mentioned above, which focused on the identification of the role of exosomal miRNA in the diagnosis of PDAC, providing evidence for the routine application of specific exosome components in the early detection of pancreatic cancer and additional information are presented in Table 1.

**Table 1:** Available exosomes involved in pancreatic cancer diagnosis

Type of cancer	Used samples during the studies	Exosomes	Application in pancreatic cancer	References
Pancreatic cancer	Exosomes were collected from the conditioned media of pancreatic cancer cell lines and plasma samples of localized pancreatic cancer patients (Stage I-IIA) healthy subjects.	miR-196a and miR-1246	Diagnosis	[57]
	The exosomes were collected from patients with pancreatic cancer, and patients without neoplasms (controls).	miR-191, miR-21, miR-451a	Diagnosis	[58]
	Plasma RNAs were extracted from pancreatic cancer patients, chronic pancreatitis patients, and healthy controls.	miRNA-16a and miRNA-196a, together with CA19–9	Diagnosis	[62]
	The plasma samples were obtained from pancreatic ductal adenocarcinoma (PDAC), intraductal papillary mucinous neoplasm (IPMN) patients, and healthy controls.	miRNA-483-3p	Diagnosis	[59]
	Saliva from pancreatobiliary tract cancer patients, healthy donors.	miR-1246 and miR-4644	Diagnosis	[66]
	Portal vein blood and peripheral blood collected from PDAC patients during curative pancreatectomy.	miR-4525, miR-451a, and miR-21	Diagnosis	[63]
	The plasma of patients with primary pancreatic ductal adenocarcinoma and plasma from healthy controls.	miR-155, miR196a	Diagnosis	[67]
	Serum from pancreatic cancer, chronic pancreatitis, and benign pancreatic tumors patients, also they used serum from non-pancreatic cancer.	miR-1246, miR-4644, miR-3976, miR-4306 and CD44v6, Tspan8, EpCAM, MET, CD104	Diagnosis	[24]
	The peripheral blood plasma of patients with PC and healthy controls.	exmiR-21	Diagnosis	[68]
	Serum, benign pancreatic tumors, ampullary carcinomas, chronic pancreatitis, healthy donors.	miR-17-5p	Diagnosis	[69]
	The plasma of pancreatic cancer, chronic pancreatitis patients, and healthy controls.	miR-10b	Diagnosis	[70]
	The exosomes have been extracted from media that contained the PANC1 cell line.	miR-550	Diagnosis	[71]
	The exosomes were collected from the blood of pancreatic cancer, chronic pancreatitis patients, normal donors.	microRNA-10b, (miR-10b), miR-21, miR-30c, and miR-181a and low miR-let7a	Diagnosis	[72]

### Exosomes are essential in pancreatic cancer treatment

Various studies highlighted that exosomes play a significant role in the treatment of pancreatic cancer by significantly reducing the progression and the aggressiveness of pancreatic cancer [73]. All points of view mentioned above are possible just because of exosomes’ ability to carry and deliver different kinds of substances that target whether cancerous cells or genes involved in pancreatic cancer development [74]. For example, one of the studies showed that, after the priming of both MSCs and Paclitaxel, considerably stopped the proliferation of CFPAC-1 pancreatic cell lines by releasing paclitaxel-having exosomes into a well-controlled cell culture medium [75]. It was also found that exosomes transfer Curcumin in pancreatic cancer cells to encourage in vitro cytotoxicity [23]. Besides, to deal with gemcitabine resistance in pancreatic cancer, Aspe JR et al. used exosomes to deliver survivin T34A to pancreatic cancer cell line (MiaPaCa-2) which in turn restored gemcitabine sensitivity in pancreatic cancer cell [76]. Using clinical trials, clinicians can determine whether new treatments are safe and effective in treating different diseases, including pancreatic cancer. Although there are not many clinical trials performed on using exosomes as drug carriers, currently, there is one clinical trial that is in its Phase I (NCT03608631). This clinical trial is using exosomes derived from mesenchymal stem cells to deliver small interference RNA that targets KrasG12D mutations in patients with metastatic pancreatic adenocarcinoma.

In cancer treatment, stopping cancer development is essential, basing on that various studies have carried out to verify whether exosomes can be used to prevent pancreatic cancer progression. For example, a recent study reported that exosomal miR-7 blocked pancreatic cancer events such as growth, migration, and invasion. Also, they noted that the overexpression of miR-7 inhibited tumor development in mice by directly targeting MAP 3 K9 [77]. Another study which had the aim of determining the role of DLC1 and microRNA-195 in pancreatic cancer, during their research, firstly, they noted that overexpression of miRNA-195 could stop proliferation, migration, and invasion of pancreatic cancer cells. Secondly, they also found that exosomal MicroRNA-195 suppressed PC development by targeting DCL1 [78]. It is also known that inhibiting some genes in combination with some exosomal microRNAs can help to inhibit cancer progression in different cancer types, including pancreatic cancer. An oncogene in pancreatic cancer known as TUG1 put together miRNA-299-3p brought a significant approach in treating pancreatic cancer [79]. The inhibition of the two significantly suppressed the Notch 1 pathway, and this could stop pancreatic cancer advancement [80]. Making some biological modifications on exosomes can also be a useful tool to produce new therapeutic novels to fight against pancreatic cancer. A study aimed at determining the curative abilities of miR-16a reported that this exosomal microRNA could inhibit some essential genes involved in cell cycle arrest and cell proliferation [67]. It has been reported that miR-15a makes PC resistant to gemcitabine, to overcome this situation, they modified the exosomal micro RNA in question to 5-FU-miR-15a, which stopped PC cell proliferation. The combination significantly suppressed pancreatic cancer lung metastatic development [81]. Pancreatic cancer is one of a malignant disease that resists to some of the available chemotherapies, as the battle continues to eliminate this burden, the researchers found that exosomes may be excellent tools to prevent this problem, for example, it has been reported that microRNA-410-3p significantly reduced the PC cancer resistance to gemcitabine by inhibiting HMGB1-mediated autophagy this made the exosomes to be considered as good candidates to be used in treating pancreatic cancer [82].

Pancreatic cancer is a cancerous disease which initiation is based on different gene mutations, such as TP53, Kras, CDKN2A [83]. Among all of those genes which are mutationally activated in PC, Kras is the most mutated gene in pancreatic cancer [84], and this made the researchers start to think that maybe targeting Kras gene pathways using exosomes could help in the treatment of pancreatic cancer. A recent study reported that an exosome that was extracted from normal fibroblast-like mesenchymal and modified to bear siRNA targeting Kras^G12D^ was able to stop pancreatic cancer progression in different GEMMs and greatly improved these mouse models overall survival rates [85]. Basing on the facts mentioned above, it is clear that exosomes significantly play an essential role in treating pancreatic cancer.

### The involvement of exosomes in pancreatic cancer TME

The tumor volume contains not just highly diverse cancerous cells groups; it also includes several essential and infiltrating host cells, secreted factors, and extracellular matrix proteins, generally referred to as the tumor microenvironment [86]. It is widely known that the tumor microenvironment is made up of macrophages, dendritic cells, T cells, endothelial cells and fibroblasts, proteases, cytokines, ECM play a significant role in tumor evolution and metastasis in different types of cancer especially in pancreatic cancer [87]. Growing pieces of evidence have demonstrated that exosomes are strongly connected to the tumor microenvironment [88]. Basing on these facts, researchers continued to explore more about the role of exosomes in the pancreatic cancer CAFs, which are essential components of the TME. It has been reported that some chemotherapies induce CAFs to start to release more exosomes, which in turn promote pancreatic cancer cell proliferation and drug resistance [89]. Exosomes also play a role as cell transformation initiator in human pancreatic cancer; a recent study reported that pancreatic cancer cell exosomes were involved in malignant cell transformation of NIH/3 T3 cells, contrarily, this was impossible on exosomes from healthy pancreatic cells or primary fibroblast cells [90]. Macrophages-delivered exosomes are one of the pancreatic cancers TME components, and they are said to be involved in different PC essential events. Yin Z et al. showed that macrophage-derived exosomal miR-501-3p suppresses the TGFBR3 gene and promotes the growth of PC by triggering the TGF-β signaling pathway [91]. As mentioned above, it is quite clear that exosomes play essential roles in pancreatic cancer TME.

### Exosomes are essential tools to monitor response to therapy in pancreatic cancer

Currently, different therapies are used to treat pancreatic cancer, and it is necessary to monitor or track the therapies’ responses. One of the most common biomarkers is CA-19-9, has been used for so long to monitor the therapeutic responses in pancreatic cancer [92].

As mentioned earlier, exosomes play different roles in pancreatic cancer events like metastasis, cell proliferation, angiogenesis, and treatment of pancreatic cancer. Besides, exosomes are also believed to play a significant role in monitoring response to therapy. For example, one study tested cell surface proteoglycan, glypican-1 (GPC1) on serum exosomes in patients harbouring pancreatic cancer at both pre-and post-operative levels. The results highlighted a substantial decrease following surgical resection, suggesting that glypican-1 plus exosomes that may function as a non-invasive biomarker and a possible tracking tool for the identification of therapy response [93]. It has also been found that exosomal forms of the EGFR released from pancreatic cancer cells can also be the right candidate to track therapy response [94]. Another study which was done on plasma exosomes reported that increased rates of miR-221 in the blood found three weeks after initiation of treatment with lapatinib and capecitabine have been related to drug resistance, this made miR-221 be a right candidate for monitoring these two therapies response in pancreatic cancer treatment [95]. Also, Miyamae M et al. also noted that the high expression of miR-744 might be a useful tool to monitor chemoresistance in pancreatic cancer [96]. Although It is clear that exosomes essential roles in controlling response to therapy, there is a need for more studies and clinical trials so that exosomes can be used as usual monitoring therapy response agents not only for pancreatic cancer but also for other types of cancer.

## Conclusions

Pancreatic cancer is one of the most lethal diseases among solid malignancies. Most patients are diagnosed with advanced or metastatic pancreatic cancer. Also, Pancreatic cancer is resistant to its routine treatments, and pancreatic cancer does not have both successful target therapies or sensitive diagnostic tools. It is essential to continue doing deep researches so that we can find for early diagnosis and establish novel therapeutic targets for pancreatic cancer. Taken together, exosomes play a significant role in intercellular communication. Several studies also confirmed that exosomes can be released by all kinds of cells and are significantly associated with multiple cancers, including pancreatic cancer. Many pieces of research proved that exosomes correlate with biogenesis, progression, metastasis, and tumor immunity in pancreatic cancer; also, exosomes are detectable in different body fluids, and that makes exosomes to be used as pancreatic cancer biomarkers. Besides, exosomes are involved in cell-cell communication, and this makes them potential candidates to be used to produce new therapeutic novels to treat not only pancreatic cancer but also other types of cancer. Although we have attempted to summarize the roles of exosomes in the treatment and diagnosis of pancreatic cancer, it is necessary to conduct in-depth researches and clinical trials so that exosomes can be used as a daily treatment and diagnostic tool for pancreatic cancer in the future.

## Data Availability

Not applicable.

## Abbreviations

PC: Pancreatic cancer
EMT: Epithelial-mesenchymal transition
TME: Tumor microenvironment
ESCRT: Endosomal sorting complexes required for transport
MAP 4 K4: Mitogen-Activated Protein Kinase Kinase Kinase 4
MAP 3 K9: Mitogen-Activated Protein Kinase Kinase Kinase Kinase 9
MAPK/ERK: Mitogen-activated protein kinase /extracellular-signal-regulated kinase
PTEN/ PI3Kγ: Phosphatase and Tensin Homolog / Phosphoinositide 3-Kinase γ
PDAC TAFs: Pancreatic ductal adenocarcinoma tumor-associated fibroblasts
HMVEC: Human microvascular endothelial cell
BTG2: B cell translocation gene 2
IPMN: Intraductal papillary mucinous neoplasm
TNM: Tumour Node Metastasis
DLC1: Deleted in liver cancer 1
TUG1: Taurine Up-Regulated 1
HMGB1: High Mobility Group Box 1
siRNA: Small interfering RNA
GEMMs: Genetically engineered mouse models
ECM: Excess extracellular matrix
CAFs: Cancer-associated fibroblasts
